# Influence of Repeated-Sprint Ability on the in-Game Activity Profiles of Semiprofessional Rugby Union Players According to Position

**DOI:** 10.3389/fspor.2022.857373

**Published:** 2022-04-25

**Authors:** Paul Glaise, Baptiste Morel, Isabelle Rogowski, Brice Cornu, Cyril Martin

**Affiliations:** ^1^Inter-University Laboratory of Human Movement Biology (LIBM EA7424), University Claude Bernard Lyon, Lyon, France; ^2^Union Sportive Bressane Pays de l'Ain (USBPA Rugby), Bourg-en-Bresse, France; ^3^Inter-University Laboratory of Human Movement Biology (LIBM EA7424), University Savoie Mont-Blanc, Chambéry, France

**Keywords:** high intensity, key performance indicators, movement characteristics, RSA, rugby

## Abstract

This study investigated the influence of repeated-sprint ability (RSA) on the activity of rugby union players in a competitive situation according to their position. Thirty-three semiprofessional rugby union players (age, 25.6 ± 4.3; height, 184.0 ± 8.0 cm; weight, 98.9 ± 13.9 kg, ~20 h training a week), divided into two position subgroups (forwards *n* = 20, backs *n* = 13) or four positional subgroups (front row and locks *n* = 13, back row *n* = 7, inside backs *n* = 6, outside backs *n* = 7), were tested. Their RSA was assessed with a 12 × 20 m sprint test over a 20 s cycle. GPS data (distance, acceleration, number of sprints, maximum velocity, and high-velocity running) and technical data were collected on 18 semiprofessional division rugby union games. In forwards, players with lower cumulated sprint time in the RSA test produced significantly more accelerations (*ρ* = −0.85, *p* < 0.001) and more combat actions per match minute (*ρ* = −0.69, *p* < 0.001). In backs, RSA was significantly correlated with high-intensity running [distance (*ρ* = −0.76), V_max_ (*ρ* = −0.84), sprints frequency (*ρ* = −0.71), high-velocity running (*ρ* = −0.76), all *p* < 0.01]. Then, the players were divided into four subgroups (front row and locks, back row, inside backs and outside backs). RSA was significantly associated with the number of accelerations (*ρ* = −0.96, *p* <001) and combat actions in front row and locks (*ρ* = −0.71, *p* = 0.007). In the back row, RSA was correlated with distance (*ρ* = −0.96, *p* = 0.003) and the frequency of combat actions (*ρ* = −0.79, *p* = 0.04). In inside backs, RSA was significantly (all *p* < 0.01) correlated with distance (*ρ* = −0.81), number of accelerations (*ρ* = −0.94) and high-velocity running (*ρ* = −0.94), while in outside backs, RSA was associated with sprint frequency (*ρ* = −0.85) and the maximal in-game velocity reached (*ρ* = −0.89). These results demonstrate that RSA is associated with match running and combat activity performance (i) regardless of the position on the pitch and (ii) specifically for each player's position by improving the corresponding activity profile.

## Introduction

Rugby union is an intermittent sport with high-intensity collisions and running evasions, leading to combat situations (maul, ruck, tackle, and impact) and running phases (Deutsch et al., 2007). Rugby requires the repetition of high-intensity force- and velocity-dependent tasks interspersed with recovery phases of random duration and intensity, which are often incomplete (Gabbett et al., 2012; Morel et al., 2015). Thus, high-intensity efforts represent an important part of the efforts in rugby union for all players (Austin et al., 2011a). Game cumulated high-intensity time represents 9–19-min for forwards and 3–7-min for backs (Roberts et al., 2008; Austin et al., 2011a). The ball in play demand is about 108.6 ± 8.5 m.min^−1^, 0.8 ± 0.2 accelerations per minute (>3 m.s^−2^) and 0.8 ± 0.3 collisions per minute (Pollard et al., 2018). On average, this represents up to ~45% of the ball-in-play time spent at intensities close to the maximal heart rate (i.e., >90%) (Cunniffe et al., 2009; Sparks and Coetzee, 2013).

In rugby union, players perform different types of maximum efforts, such as sprints, accelerations, tackles, and rucks. Those activity types are highly dependent on the player's position (Austin et al., 2011a) at any level of competition (Takamori et al., 2020; Fornasier-Santos et al., 2021). Most studies divide the players into two positional groups: forwards and backs. Forwards are generally divided into more specific positional roles such as front row, locks, and back row (Zabaloy et al., 2021). Forwards are generally involved in combat actions, such as scrums, tackles or rucks, requiring a maximum or near maximum intensity (Cunniffe et al., 2009). Their total running distance is lower compared to backs (Doutreloux et al., 2002; Roberts et al., 2008; Cunniffe et al., 2009; Austin et al., 2011a; Gabbett et al., 2012; Sparks and Coetzee, 2013; Morel et al., 2015). Thus, forwards produce more repeated high-intensity exercise (RHIE; >3 maximum-intensity efforts interspersed with <21 s rest periods assessed by time-motion analysis) (Austin et al., 2011b, 2013) than backs. Jones et al. (2015) identified the key performance indicators (KPI) in forwards being the number and efficiency of combat actions including tackles, rucks and duels and intense accelerations (Jones et al., 2006). However, among forwards, the activity of the front row and locks differs from that of the back row (Austin et al., 2011a, 2013); total distance and high intensity distance in back row has been shown to be higher than in front row and locks (Cahill et al., 2013).

In-match demands for the backs are different since they complete more high-intensity distance runs (7.4 m.min-1 above 18–20 km·h^−1^) (Austin et al., 2011b, 2013; Coughlan et al., 2011; Suárez-Arrones et al., 2012), high accelerations (5.7+3 accelerations >3 m.s^2^) (Cunningham et al., 2016) and high-velocity running (>20 km·h^−1^) (Doutreloux et al., 2002; Coughlan et al., 2011; Gabbett et al., 2012; Suárez-Arrones et al., 2012) and reach a higher maximum velocity than the forwards (26.3 vs. 30.2 km·h^−1^ median maximum velocity in forwards compared to backs) (Cahill et al., 2013). In this sense, Backs are generally categorized into two subgroups (inside backs and outside backs), whose activity has also been shown to differ (higher sprint distance and velocity in outside backs compared with inside back) (Cahill et al., 2013).

Repeated-sprint ability (RSA) is defined as the ability to reproduce maximum or near-maximum intensity in brief efforts (<10 s), interspersed with short and incomplete recovery periods (usually <90 s) (Girard et al., 2011). In team sports such as rugby union, the ability to recover and repeat sprints is a fundamental performance factor (Bishop et al., 2011). Better RSA is related, in rugby league, to a greater distance traveled at high velocity for all players (Gabbett et al., 2013), as well as, in rugby union, to higher activity ratio (i.e., the number of actions per time unit) and number of successful tackles in forwards (Smart et al., 2014). Moreover, a better RSA has been shown to be associated with higher RHIE in rugby league (Gabbett et al., 2013).

To date, only two studies have examined the relationship between RSA and performance in rugby competition. The first study examined the relationship between RSA and technical performance in forwards and backs but did not analyze physical match performance (Smart et al., 2014). The second study focused on rugby league players and determined the relationships between RSA and physical match performance, without distinguishing the playing positions (Gabbett et al., 2013).

As suggested above, the activity profile in rugby union is highly dependent on position. Thus, it is crucial to identify whether RSA is related to key performance indicators (KPI), including moving (Distance, accelerations, high-speed running and sprints) and fighting situations (number of collision situations), according to more specific playing positions in rugby union. Therefore, the objective of this study was to analyze the relationship between RSA and game performance in competition according to position and to consider the KPI specific to each position, as defined above. We hypothesized that RSA would be correlated with KPI during competition games and associated with different KPI depending on playing position.

## Methods

### Subjects

Thirty-three semiprofessional male rugby union players (age, 25.6 ± 4.3; height, 184.0 ± 8.0 cm; weight, 98.9 ± 13.9 kg; 4 skinfolds body fat, 16.8 ± 3.4%), competing in a semiprofessional division, were included in this study and divided into two groups: 20 forwards (height, 187.4 ± 8.0 cm; weight, 108.1 ± 7.9 kg; 4 skinfolds body fat, 18.1 ± 3.5%) and 13 backs (height, 178.8 ± 4.6 cm; weight, 85,5 ± 6.6 kg; 4 skinfolds body fat, 14.8 ± 2.1%). The players achieved a training volume of ~20 h.week^−1^ (eight rugby sessions, four strength and velocity development sessions, one aerobic conditioning development session, and one match).

### Study Design

A retrospective analysis was used to study GPS and technical data from 18 consecutive games (game 3 to game 21 of the 2019–2020 Federal 1 Season) and physical test results (30-m sprint time, RSA, and 30–15 intermittent fitness test). These tests were performed in the middle of the championship (on the weeks of games 8, 9, and 10, one test per week) under dry conditions with an average temperature of 13°C over 3 weeks in November. All the tests were performed in the early afternoon. The 30–15 intermittent fitness test (30–15 IFT) and the 30-m sprint test were performed to put into perspective the results obtained for the RSA. The 30 m sprint test was realized in the first week, then RSA test in the next week and 30–15 IFT in the third week. During test weeks, the training load was reduced to account for assessment tests in the overall load, and ensure that players did not train harder than normal (the month-average training load, monitored by Borg's CR10 scale from September to December (10,844 ± 1,916 A.U. [arbitrary units]),was not significantly different (*p* = 0.84) to the month average of the testing period (11,225 ± 1,272 A.U. [arbitrary units]), and the players were instructed not to consume caffeine 48 h before the tests. All these data were routinely collected by the staff to guide the training program and quantify the training load. The study received institutional ethics approval and used safe procedures for the participants. All players who participated in at least 10 games were included after giving their informed consent. Physical test performances were correlated with GPS and performance data according to position, with (i) a classification within two groups: forwards (*n* = 20) and backs (*n* = 13) and (ii) a classification into four groups: front row and locks (FRL, *n* = 13), back row (*n* = 7), inside backs (*n* = 6) and outside backs (*n* = 7), as used in previous studies (Deutsch et al., 2007; Austin et al., 2011b).

### 30-M Sprint Test

The 30-m sprint test also was performed around match nine, during the first training sequence of the day. After a standardized warm-up (5-min of dynamic stretching and joint mobilization exercises, 10-min of athletic skills and moderate-intensity running, followed by 10, 15, and 20-m accelerations each repeated twice), the athletes were given three trials, with 5 min recovery intervals in between, to perform the 30-m sprint as quickly as possible. The times were recorded using photoelectric cells (Brower Timing Systems, Draper, UT, USA) positioned at the start and at 30-m. The lowest time was considered for further analysis.

### Repeated-Sprint Ability

After a standardized warm-up (same as the 30-m sprint test), the RSA was measured on artificial turf and in no-wind conditions at 10 am as follows: (i) two maximal 20-m sprints were performed with a full recovery (2 min between each), and the best time was assigned as a reference, (ii) 5-min of recovery were allowed before the beginning of the test, and (iii) 12 × 20-m sprints every 20-s were performed at maximal intensity (Gabbett et al., 2011). The first two 12 × 20-m sprints were controlled in real time to ensure that the participant performed the sprints close to his maximum intensity (for three athletes for whom the first two sprints did not reach a threshold of 95% of the reference time, the test was stopped and restarted after 5-min of passive recovery). During all sprints, the participants received strong verbal encouragement. The times were measured using photocells (Brower Timing Systems, Draper, UT, USA) positioned at the start and at 20-m. The RSA_tot_ (sum of the 12 sprint times) and the performance decrement score (RSA_dec_, Equation 1) were calculated and considered as RSA indices.


(1) $$\text{RSAdec}=(\;\frac{\left(S1+S2+S3\ldots +S12\right)}{12*Sbest\;}-1)*100$$

**Equation 1. Performance decrement score**. *RSA*_*dec*_ is the percentage decrease in performance, *S*_*n*_ is the time of the *n* sprint and *S*_*best*_ is the time of the best sprint achieved (Girard et al., 2011).

### 30–15 Intermittent Fitness Test

After a standardized warm-up (5-min of muscular work followed by 10-min of athletic drills and running at low and medium intensity), the 30–15 Intermittent Fitness Test (IFT) (Buchheit, 2008a) was performed during the first training sequence of the day around game 9, using natural turf. The 30–15 (IFT) is an incremental test consisting of 40-m shuttle bouts of 30-s at a given velocity, interspersed with 15-s of passive recovery. Each stage is followed by a velocity increment of 0.5 km·h^−1^, marked by an increase in the frequency of sound signals (audible signals allow participants to run 40-m shuttles at the given velocity). The test was stopped when the player could no longer maintain the required running speed. The 30–15 IFT end-running velocity (VIFT) recorded corresponded to the velocity reached during the last step fully completed by the player.

### In-Game GPS Data

The GPS system data were collected during 18 matches in the French semiprofessional third division (Federale 1). The time and position were recorded by a GPS unit (sampling rate, 10 Hz; Catapult Innovations, Melbourne, Australia), fixed between the scapulas. Accelerations were recorded with the GPS-integrated triaxial accelerometer (sampling rate: 400 Hz). Distance per minute (D·min^−1^, m.min^−1^), maximum velocity (V_max_, km·h^−1^), sprint frequency (S·min^−1^: number of sprints >25 km·h^−1^ per minute), distance traveled in high-velocity running zone per minute (HSR·min^−1^: sprint zone corresponds to velocity >25 km·h^−1^) and the number of high accelerations per minute (A·min^−1^: number of accelerations > 3 m.s^−2^) (Jones et al., 2015) were obtained from the software provided by the manufacturer (Catapult, playertek + V1.0.3). Only data from players who participated in at least 30 min on the field for each game were included.

### Game Technical Statistics

During the match, individual player activities were recorded by video analysis (Sportscode, Jeddah, Saudi Arabia). The number of combat situations (tackle, duel, ruck, maul, and scrum) related to the player's playing time on the field was monitored for each participant and noted Na·min^−1^. The tackle count represents the number of tackles attempts (missed and succeeded). The duel count represents the number of running evasion events attempted with the ball in hand. The maul count represents the number of offensive and defensive maul participations. Ruck is the number of participations in offensive and defensive rucks. The technical efficiency of tackle (TackleEFF), and ruck (RuckEFF) were calculated as the percentage of successful actions relative to total actions attempted by a player and were coded a single rater to avoid inter rater variation. Efficiency was based on objective criteria defined and used by two professional coaches to quantify the team's performance in season (Supplemental Data 2). This method has been proven to be more reliable in quantifying collisions than microtechnology analysis (Reardon et al., 2017). The intra observer variation was checked using an intra-class correlation coefficient for rucking efficiency (ICC = 0.97 [0.94; 0.99]; SEM = 0.025) and tackling efficiency (ICC = 0.98 [0.97; 0.96]; SEM = 0.023). The inter-subject coefficients of variation were 0.13 and 0.22 % for RuckEFF and TackleEFF, respectively. The mean intrasubject coefficients of variation were 0.11 ± 0.08% for Tackle EFF and 0.09 ± 0.03% for Ruck EFF.

### Statistical Analysis

All data are expressed as means + SD. The Shapiro-Wilk test and Levene's test were applied to verify the normality and homoscedasticity of the variables. When these two parameters were verified, an ANOVA test was used to determine intergroup differences, followed by a Tukey *post hoc* test when appropriate. When one of the two parameters was not verified, we applied non-parametric tests: Kruskal-Wallis ANOVA and the Mann-Whitney U test were used to account for intergroup differences. Partial η^2^ (0.01, 0.06 and 0.14 for small, medium and large effect, respectively) or Cohen's d (0.2, 0.5, and 0.8 for small, medium and large effect, respectively) effect sizes were calculated and expressed as an absolute value to determine the magnitude and practical relevance of changes. Spearman correlations were determined to identify relationships among the selected variables. All calculations were performed using Statistica 13.2 software (StatSoft, Inc., Tulsa, USA) with a threshold of significance set at *p* ≤ 0.05.

## Results

**Figure 1:**
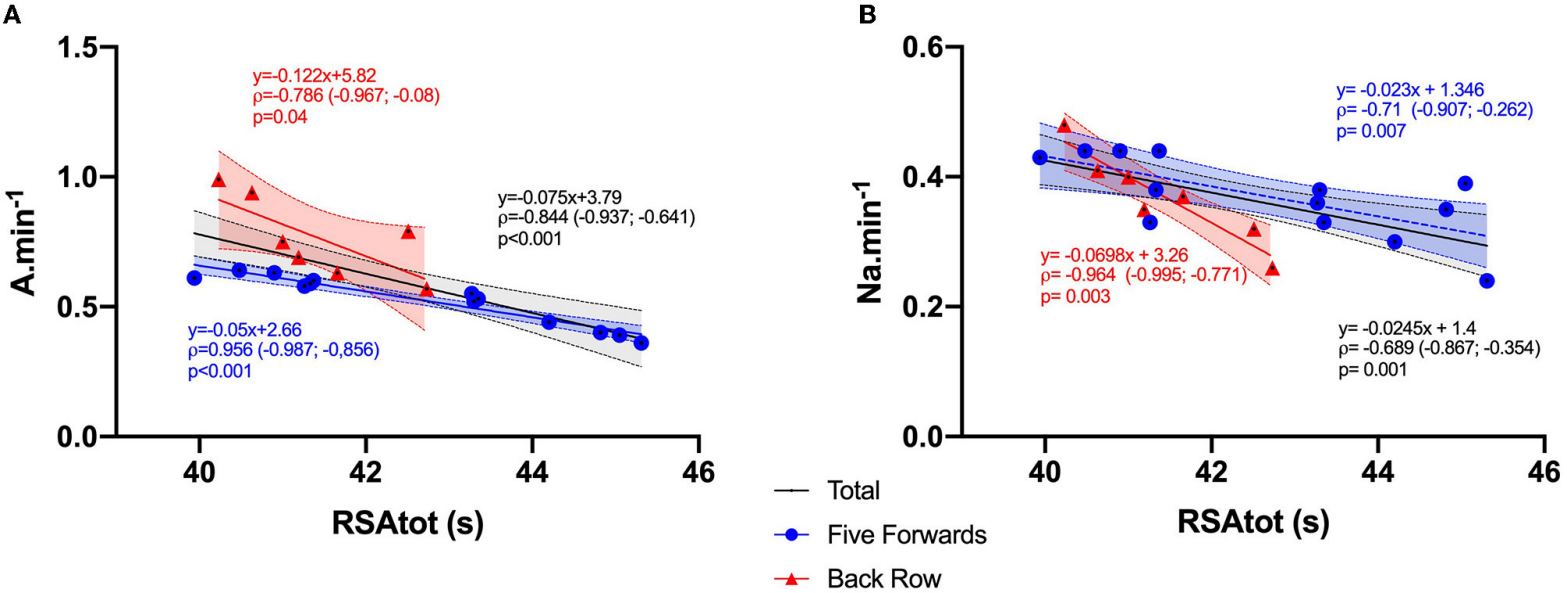
Relationships between physical abilities and player movement characteristics in game in forwards: **(A)** Relationships between RSA_tot_ and acceleration number per minute (A·min^−1^) in forwards; **(B)** Relationships between RSA_tot_ and combat action number per minute (Na·min^−1^) in forwards.

In forwards (Figure 1 and Table 1), while RSA_dec_ was not significantly related to player movement characteristics, RSA_tot_ was significantly correlated with V_max_, S·min^−1^, A·min^−1^ and HSR·min^−1^ (*p* = 0.02, *p* = 0.05, *p* < 0.01 and *p* = 0.047, respectively). Additionally, VIFT was associated to movement characteristics across all variables (All *p* < 0.05). Thirty-meter sprint time was correlated with A·min^−1^ (*p* = 0.009). When players were divided into four groups, there was a strong negative correlation in FRL between RSA_tot_ and A·min^−1^ (*ρ* = −0.96, *p* < 0.001), and VIFT was significantly correlated with HSR·min^−1^ (*p* = 0.008) and S·min^−1^ (*p* = 0.027). The 30-m sprint time was inversely correlated with A·min^−1^ (*p* = 0.002). In the back row, a better performance in RSA_tot_ was associated with a higher D·min^−1^ (*ρ* = −0.96, *p* = 0.002) and A·min^−1^ (*p* = 0.04).

**Table 1:** Relationships between physical abilities and movement characteristics in matches according to position.

		**30 m sprint time**	**VIFT**	**RSAtot (s)**	**RSAdec (%)**
		**(s)**	**(km·h^**−1**^)**		
Forward	D·min^−1^ (m.min^−1^)	−0.26	0.62*	−0.36	0.07
	Vmax (km·h^−1^)	−0.36	0.77*	−0.46*	−0.11
	S·min^−1^	−0.41	0.79*	−0.50*	−0.03
	A·min^−1^	−0.69*	0.81*	−0.85*	−0.14
	HSR·min^−1^	−0.36	0.73*	−0.48*	−0.07
Back	D·min^−1^ (m.min^−1^)	−0.15	0.36	−0.69*	0.01
	Vmax (km·h^−1^)	−0.54	0.51	−0.84*	0.05
	S·min^−1^	−0.47	0.50	−0.71*	−0.07
	A·min^−1^	0.12	−0.10	−0.28	0.27
	HSR·min^−1^	−0.50	0.42	−0.76*	−0.01
Front row and locks	D·min^−1^ (m.min^−1^)	−0.04	0.30	0.01	0.30
	Vmax (km·h^−1^)	−0.27	0.48	−0.31	0.27
	S·min^−1^	−0.40	0.56*	−0.41	0.25
	A·min^−1^	−0.80*	0.55	−0.96*	0.04
	HSR·min^−1^	−0.35	0.59*	−0.34	0.25
Back row	D·min^−1^ (m.min^−1^)	−0.75	0.40	−0.96*	0.32
	Vmax (km·h^−1^)	0.18	0.09	−0.01	0.11
	S·min^−1^	0.07	0.15	−0.04	0.64
	A·min^−1^	−0.32	0.76*	−0.79*	0.18
	HSR·min^−1^	0.36	0.26	0.01	0.29
Inside backs	D·min^−1^ (m.min^−1^)	−0.77	0.26	−0.81*	−0.26
	Vmax (km·h^−1^)	−0.83*	0.41	−0.77	−0.20
	S·min^−1^	0.09	0.47	−0.66	−0.09
	A·min^−1^	−0.09	0.15	−0.94*	−0.08
	HSR·min^−1^	−0.09	0.15	−0.94*	−0.09
Outside backs	D·min^−1^ (m.min^−1^)	−0.54	0.51	−0.29	0.11
	Vmax (km·h^−1^)	0.10	0.34	−0.89*	0.07
	S·min^−1^	−0.25	0.58	−0.85*	−0.21
	A·min^−1^	0.14	−0.50	−0.67	0.29
	HSR·min^−1^	−0.43	0.58	−0.60	0.01

*Values represent Spearman's ρ. VIFT, 30-15 IFT end-running velocity; RSAtot, total time for repeated-sprint ability; RSAdec, performance decrement percentage; D·min^−1^, distance per min (m.min^−1^); Vmax, maximum velocity reached in game (km·h^−1^); S·min^−1^, number of sprints per minute; A·min^−1^, number of accelerations per minute; HSR·min^−1^, high-velocity running per minute*.

* *p < 0.05*.

*Bold values means significant correlations (p < 0.05)*.

**Figure 2:**
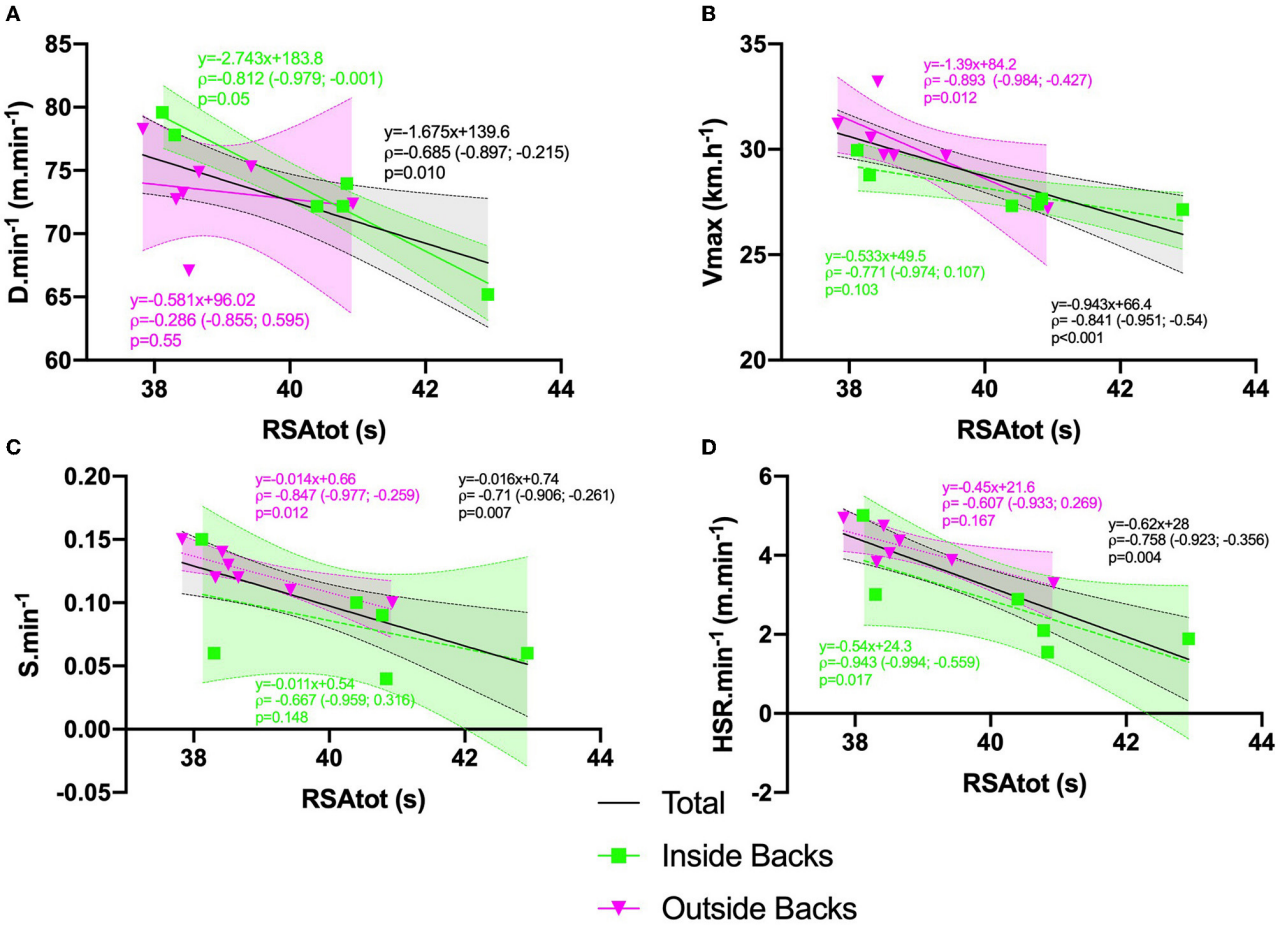
Relationships between physical abilities and player movement characteristics in game in backs: **(A)** Relationships between RSAtot and distance per minute (D·min^−1^) in backs; **(B)** Relationships between RSAtot and maximal velocity (Vmax; km·h^−1^) in backs; **(C)** Relationships between RSAtot and sprint frequency (S·min^−1^) in backs; **(D)** Relationships between RSAtot and high-velocity running distance (HSR·min^−1^; m.min^−1^) in backs.

In backs (Figure 2 and Table 1), RSA_tot_ was negatively correlated with D·min^−1^, V_max_, S·min^−1^ and HSR·min^−1^ (*p* = 0.0.01, *p* < 0.001, *p* = 0.007, and *p* = 0.004, respectively), whereas no significant relationship was found between player movement characteristics and 30-m sprint time, VIFT or RSA_dec_. When players were divided into four groups, while RSA_dec_ and VIFT were not significantly related to player movement data in inside backs, strong negative correlations were found (*ρ* = −0.81, *p* = 0.05; ρ = −0.94, *p* = 0.033, and ρ = −0.94, *p* = 0.017) between RSA_tot_ and D·min^−1^, A·min^−1^, and HSR·min^−1^, respectively. In outside backs, RSA_tot_ was negatively associated to S·min^−1^ (*ρ* = −0.84, *p* = 0.012) and V_max_ (*ρ* = −0.89, *p* = 0.012).

### Relationships Between Physical Abilities and Technical Performance in Matches (Figure 1 and Table 2)

In forwards, lower time in RSA_tot_ was associated with higher Na·min^−1^ (*p* = 0.001). Moreover, there was a significant correlation between VIFT and TackleEFF (*p* = 0.028) and between 30-m sprint time and Na·min^−1^ (*p* = 0.007). When players were divided into positional subgroups, RSA_tot_ and 30-m sprint time were negatively associated with Na·min^−1^ (both *p* = 0.007), and VIFT was positively correlated with TackleEFF (*p* = 0.05) in FRL. In the back row, a strong association was found between RSA_tot_ and Na·min^−1^ (*ρ* = −0.96, *p* = 0.002), and VIFT was correlated with RuckEFF (*p* = 0.047).

**Table 2:** Relationships between physical abilities and technical performance in matches according to position.

		**30 m sprint time (s)**	**VIFT (km·h^**−1**^)**	**RSAtot (s)**	**RSAdec (%)**
Forward	Na·min^−1^	−0.62*	0.24	−0.69*	−0.02
	RuckEFF	−0.01	0.18	−0.06	−0.13
	TackleEFF	−0.05	0.50*	−0.17	−0.28
Back	Na·min^−1^	0.10	−0.17	0.01	0.02
	RuckEFF	0.10	0.02	0.49	−0.09
	TackleEFF	0.43	−0.01	−0.25	−0.21
Front row and locks	Na·min^−1^	−0.70*	0.45	−0.65*	0.01
	RuckEFF	0.01	0.18	−0.10	−0.06
	TackleEFF	0.03	0.64*	−0.18	−0.30
Back row	Na·min^−1^	−0.57	0.42	−0.96*	0.14
	RuckEFF	0.03	0.76*	−0.14	−0.04
	TackleEFF	−0.34	−0.20	−0.20	0.36
Inside backs	Na·min^−1^	−0.37	−0.38	−0.60	0.31
	RuckEFF	0.60	0.38	0.26	0.09
	TackleEFF	0.49	−0.26	0.60	−0.49
Outside backs	Na·min^−1^	−0.46	0.24	−0.71	−0.50
	RuckEFF	−0.11	0.09	−0.46	−0.21
	TackleEFF	−0.14	0.37	−0.40	0.07

*Values represent Spearman's ρ. VIFT, 30-15 IFT end-running velocity; RSAtot, total time for repeated-sprint ability; RSAdec, performance decrement percentage; Na·min^−1^, number of combat actions per minute; RuckEFF, ruck efficiency ratio; TackleEFF, tackle efficiency ratio*.

* *p < 0.05*.

*Bold values means significant correlations (p < 0.05)*.

For backs, inside backs and outside backs, no significant relationships were observed between physical abilities and technical performance. Differences in physical ability and activity data in the matches according to position are shown in Supplemental Datas 3, 4, respectively.

## Discussion

The aim of this study was to investigate the relationships between RSA and several match performance parameters according to position in rugby union. The main results of this study show significant correlations between RSA and players' movement activity and game-based performance variables in rugby union match. The main finding is that for each playing position, RSA performance was significantly associated with specific KPI in matches, including specific moving and fighting situations.

Previous studies have investigated the relationship between RSA and movement characteristics and technical data on the entire team (Gabbett et al., 2013) and on the differences between forwards and backs (Smart et al., 2014). The purpose of this study was to go beyond these classifications as there are highly specific tasks, duty cycle, etc. in each of these group depending on the position (Austin et al., 2011a, 2013; Cahill et al., 2013); thus, this study is the first to analyze movement characteristics and technical data in the same research, with a refined analysis based on 4 position subgroups.

The better the performance in RSA_tot_ was, the more accelerations and combat situations the forwards performed. It has been reported that the number of tackles and the number of offensive tackles had a positive impact on match performance, particularly for forwards, and that forwards have a higher activity index (number of actions per minute of ball in play) when matches are won, while movement activity (GPS data) was described as a positive performance indicator for backs (Dubois et al., 2020). Previous research showed that RSA was associated with the number of total collisions (which was associated to maximal aerobic power) and high-velocity distance in players with a higher fitness level in the rugby league. VIFT has previously been associated with maximum aerobic speed and maximal oxygen consumption (Buchheit, 2008b; Buchheit et al., 2009). The significant correlations observed between VIFT and player movements are consistent as higher maximal aerobic power will allow players to have produced a greater work on a match scale.

Furthermore, researchers demonstrated that mean sprint time was related to activity rate, successful tackles and successful jackals (stealing the ball from the tackle) in forwards (Smart et al., 2014). In addition, the winning teams in rugby league had players who performed significantly more RHIE than losing teams (Gabbett, 2013). The importance for all players to repeat high-intensity efforts may be similar in rugby union. This capacity to reproduce high-intensity efforts could diminish at the end of the game independently of the position (Beard et al., 2019; Fornasier-Santos et al., 2021) and could depend on the player's status, especially for forward starters who tend to lose performance during the game (Tee et al., 2020). In addition, it has been shown that RSA performance is an important physical ability for selection in the rugby league (Le Rossignol et al., 2014). Our results thus confirm that RSA could be a key physical ability for forward players to perform during matches.

However, when analyzing FRL and Back row separately, relationships between RSA and movement characteristics appear to differ slightly. It has been shown in previous studies that FRL players completed more RHIE (11-18 RHIE per game) than full backs (Austin et al., 2011b) and the highest number of accelerations (Donkin et al., 2020), RSA_tot_ was significantly and inversely correlated with Na·min^−1^ and A·min^−1^. In this regard, the latter study (Cunningham et al., 2016) suggests that RSA performance is an important indicator of the ability to reproduce the high-intensity efforts that props, hookers and second row players are confronted with. Likewise, VIFT also was related to S·min^−1^ and HSR·min^−1^, reflecting the indispensable nature of O_2_ consumption for improving the velocity of phosphocreatine resynthesis between high-intensity efforts (Buchheit, 2012).

In back row players, RSA_tot_ was inversely correlated with D·min^−1^, A·min^−1^, and Na·min^−1^, while VIFT was related to ruckEFF. Back rows have a mixed activity pattern with high distance running, but also are largely solicited on combat situations (Cahill et al., 2013), as they perform the most RHIE (Jones et al., 2015). These results suggest that RSA also is a determining physical ability for the mixed performance of the back row, since it may play an important role on improving both their ability to produce high-mean velocity running and their number of participations in combat actions during matches.

In Backs, RSA_tot_ was correlated to all movement characteristics (except accelerations). The same correlations were not similarly observed when analyzing inside backs and outside backs separately. For inside backs, RSA was associated to total and high-velocity running distance, and number of accelerations, while strong associations were found between RSA_tot_ and V_max_ and the number of sprints in outside backs. No relationships were found between RSA and the number or efficiency of combat tasks in both subgroups. Previously authors (Austin et al., 2011b), reported that inside backs have a high level of activity since they perform ~16 ± 2 RHIE per game and cover the longest total distance (Cahill et al., 2013). Conversely, outside backs produce the highest velocity peaks (31.7 km·h) and spend twice as much distance in sprint intensity as the centers (Austin et al., 2013). Outside backs are less confronted with the repetition of high-intensity efforts, with 7 ± 3 RHIE by game (Austin et al., 2011b) due to the characteristic activity profile based on a work-recovery ratio geared toward recovery, which makes full backs less exposed to the repetition of intense efforts (Doutreloux et al., 2002). Nevertheless, a better RSA may help outside backs to increase the number of sprints and high-velocity activity.

In this study, RSA_dec_ was not associated with any of the measured game data. RSA_dec_ has previously been shown to correlate with aerobic fitness (Da Silva et al., 2010), or not (Bishop and Edge, 2006). One may hypothesize that the lack of correlation between RSA_dec_ and movement characteristics could be explained by the level of aerobic fitness of the athletes; indeed, Rodriguez-Fernandez et al. showed that RSA_dec_ correlated with aerobic abilities in soccer players with the lowest aerobic fitness, but not in those with high aerobic fitness (Rodriguez-Fernandez et al., 2019). In this study, VIFT was not correlated with RSA_dec_ in backs (*r* = 0.39, *p* = 0.17) as well as in forwards (*r* = −0.23, *p* = 0.32). Thus, RSA_dec_ might not be a robust and/or discriminant performance indicator in high level rugby union players.

The main limitations of this study are related to the small sample size. Indeed, the injuries and inclusion criteria did not allow obtaining large groups. This limitation would be problematic if no results were significant, but even with small numbers, very significant relationships with large Rs were observed. Nevertheless, one cannot exclude the possibility that some other significant correlations may have occurred with larger subgroups sample sizes, and that false-positive relationships may have occurred. On the other hand, only one RSA measure was performed over the period, which raises the question of individual variability during the season. However, familiarization with the RSA test was performed prior to the start of the study, and no significant difference between the RSA test included in this study and the familiarization test was observed (data not shown). In addition, the variability among matches might be considered a limitation that could have limited associations with fitness variables.

## Practical Applications

The findings of our research demonstrate the importance of RSA (which attests to the ability to reproduce high-intensity efforts) to match running and combat activity performance for each player's position on the pitch. A higher level of RSA is associated with improved match performance on position-specific KPI. Our results thus highlight the importance of assessing and developing RSA and, more generally, the ability to reproduce high-intensity efforts as a major performance factor for in-game specific KPI. However, these results must be confirmed by further studies, as they were obtained from analysis based on a single team and thus may have been influenced by the team's tactical and strategic characteristics (Ungureanu et al., 2021).

## Conclusion

These results demonstrate that RSA is associated with match running and combat activity performance (i) in a global way and (ii) specifically for each player's position. RSA seems to be a predominant physical quality for the performance of all positions in rugby union as it is associated with KPI, despite the great differences in activity among positions.

## Data Availability Statement

The raw data supporting the conclusions of this article will be made available by the authors, without undue reservation.

## Ethics Statement

The studies involving human participants were reviewed and approved by Local Ethics Committee, University of Lyon. The patients/participants provided their written informed consent to participate in this study.

## Author Contributions

PG and CM contributed to conception and design of the study. PG, IR, and CM organized the database and performed the statistical analysis. PG wrote the first draft of the manuscript. IR, BM, and BC wrote sections of the manuscript. All authors contributed to manuscript revision, read, and approved the submitted version.

## Funding

This study was realized within the framework of a CIFRE agreement thesis and was supported by the National Association for Research and Technology.

## Conflict of Interest

The authors declare that the research was conducted in the absence of any commercial or financial relationships that could be construed as a potential conflict of interest.

## Publisher's Note

All claims expressed in this article are solely those of the authors and do not necessarily represent those of their affiliated organizations, or those of the publisher, the editors and the reviewers. Any product that may be evaluated in this article, or claim that may be made by its manufacturer, is not guaranteed or endorsed by the publisher.
