# Supplementary material for: Influence of Repeated-Sprint Ability on the in-Game Activity Profiles of Semiprofessional Rugby Union Players According to Position
Source: Front Sports Act Living. 2022 Apr 25;4:857373. doi: 10.3389/fspor.2022.857373 (PMC9082549; doi:10.3389/fspor.2022.857373)
Supplement: Supplementary file 1 [file Data_Sheet_1.pdf]

Supplemental Data 1: Anthropometric characteristics of subjects according to position

	Total forwards N=20	Total backs N=13	ANOVA	Forwards: Front row and locks N=13	Forwards: Back row N=7	Backs: Inside backs N=6	Backs: Outside backs N=7	ANOVA
Height (cm)	187.4± 8.0 *	178.8± 4.6	$F(1,31)=12.14$, $p=0.001$, $\eta^2=0.281$	186.1± 9.4 ^{b}	189.9± 3.7 ^{b}	175.0± 3.6	182.1± 2.1	$F(3,29)=6.27$, $p=0.002$, $\eta^2=0.394$
Weight (kg)	108.1± 7.9 *	85,5± 6.6	$F(1,31)=75.23$, $p<0.001$, $\eta^2=0.708$	111.5± 7.1 $^{b, c, d}$	101.9± 5.7 $^{b, c, d}$	81.9± 8.3	87.0± 5.1	$F(3,29)=36.16$, $p<0.001$, $\eta^2=0.828$
Body fat (%)	18.1± 3.5 *	14.8± 2.1	$F(1,31)=9.66$, $p=0.004$, $\eta^2=0.238$	19.6± 9.4 $^{b, c, d}$	15.4± 3.7	15.6 ±2.5	13.9± 1.5	$F(3,29)=8.42$, $p<0.001$, $\eta^2=0.789$

* Significantly different from Total backs (p<0.05); *a* significantly different from front row and locks (p<0.05); *b* significantly different from back row (p<0.05); *c* significantly different from inside backs (p<0.05); *d* significantly different from outside backs (p<0.05)
