# Supplementary material for: Influence of Repeated-Sprint Ability on the in-Game Activity Profiles of Semiprofessional Rugby Union Players According to Position
Source: Front Sports Act Living. 2022 Apr 25;4:857373. doi: 10.3389/fspor.2022.857373 (PMC9082549; doi:10.3389/fspor.2022.857373)
Supplement: Supplementary file 2 [file Data_Sheet_2.pdf]

**Supplemental data 2**. Criteria used for assess technical efficiency of different skill of rugby union players.

| Skill | Efficiency criteria |
|---|---|
| *Duel* | Gain of advantage line, evasion , break, with the ball in hand |
| *Tackle* | Tackle completed in all direction, offensive tackle, gain of the ball, as first and second tackler |
| *Ruck* | Gain of ball, winning collision, gain of space, keep the ball safe |
