# Supplementary material for: Influence of Repeated-Sprint Ability on the in-Game Activity Profiles of Semiprofessional Rugby Union Players According to Position
Source: Front Sports Act Living. 2022 Apr 25;4:857373. doi: 10.3389/fspor.2022.857373 (PMC9082549; doi:10.3389/fspor.2022.857373)
Supplement: Supplementary file 3 [file Data_Sheet_3.pdf]

Supplemental data 2: Physical abilities according to the position.

	Total forwards N=20	Total backs N=13	ANOVA	Forwards: Front row and locks N=13	Forwards: Back row N=7	Backs: Inside backs N=6	Backs: Outside backs N=7	ANOVA
RSAtot (s)	42.2± 1.6*	39.5± 1.5	$F(1,31)=22.12$, $p<0.001$, $\eta^2=0.416$	42.6± 1.9	41.4± 0.9	40.2± 1.8 [a]	38.9± 1.0 [a, b]	$F(3,29)=9.99$, $p<0.001$, $\eta^2=0.508$
RSAdec (%)	6.2± 2.9	7.3± 2.2	$F(1,31)=1.36$, $p=0.252$, $\eta^2=0.042$	6.7± 3.3	5.3± 2.1	7.3± 2.6	7.3± 1.9	$F(3,29)=0.84$, $p=0.481$, $\eta^2=0.080$
VIFT ($km.h^{-1}$)	18.0± 1.1*	19.2± 0.9	$F(1,31)=9.86$, $p=0.004$, $\eta^2=0.241$	17.4± 0.5	19.1± 0.9 [a]	19.0 ±1.0 [a]	19.3± 0.9 [a]	$F(3,29)=11.01$, $p<0.001$, $\eta^2=0.532$
30m sprint time (s)	4.7± 0.4*	4.4± 0.2	$F(1,31)=11.49$, $p=0.002$, $\eta^2=0.270$	4.8± 0.4	4.6± 0.1	4.5± 0.1	4.2± 0.1 [a]	$F(3,29)=5.77$, $p=0.003$, $\eta^2=0.374$
Sbest (s)	3.3 ± 0.2*	3.1 ± 0.1	$F(1,31)=22.879$, $p<0.001$, $\eta^2=0.409$	3.3 ± 0.2	3.3 ± 0.1	3.1 ± 0.1 [a]	3.0 ± 0.1 [a, b]	$F(3,29)=8.76$, $p<0.001$, $\eta^2=0.459$

* Significantly different from Total backs (p<0.05); *a* significantly different from 5 forwards (p<0.05); *b* significantly different from back row (p<0.05); *c* significantly different from inside backs (p<0.05); *d* significantly different from outside backs (p<0.05)

Differences in physical abilities according to position

Backs had significantly better performance in the RSA test, with a 6.5% lower total time (RSA_{tot}) than the forwards (p < 0.001; d=1.68). RSA_{tot} was significantly lower in the outside backs group compared to the front row and locks (p < 0.001; d=2.32). and back row (p=0.02; d=2.59) and in inside backs compared to front row and locks (-2.38 s, p < 0.02; d=1.32). RSA_{dec} did not significantly differ among the positions. Backs had significantly lower best sprint time in RSA test than forwards (p < 0.01; d=1.63). Outside backs had the lower best sprint time than both groups of forwards (p < 0.01; d=2.12, p = 0.01; d=2.50 respectively compared to five forwards and back row) while inside backs produced the faster sprint compared than five forwards (p= 0.01; d=1.32).

30-15(IFT) end-running velocity was 6.5% higher in backs compared to forwards (p =0.004; d=1.12). Front row and locks had significantly lower VIFT than the other subgroups (all p < 0.009; all d > 1.72). The 30-m sprint time was significantly higher in the forwards compared to the backs (p =0.002; d=1.21). Outside backs were significantly faster (-11.3%) than front row and locks (p < 0.001; d=1.56).
