# Supplementary material for: Influence of Repeated-Sprint Ability on the in-Game Activity Profiles of Semiprofessional Rugby Union Players According to Position
Source: Front Sports Act Living. 2022 Apr 25;4:857373. doi: 10.3389/fspor.2022.857373 (PMC9082549; doi:10.3389/fspor.2022.857373)
Supplement: Supplementary file 4 [file Data_Sheet_4.pdf]

Supplemental Data 4: Performances in match game according to the position.

	Total forwards N=20	Total backs N=13	ANOVA	Forwards: Front row and locks N=13	Forwards: Back row N=7	Backs: Inside backs N=6	Backs: Outside backs N=7	ANOVA
D.min ($m.min^{-1}$)	64.2± 3.7 *	73.4± 4.1	$F(1,31)=45.47$, $p<0.001$, $\eta^2=0.595$	62.8± 3.7	66.6± 2.5	73.5± 5.1 [a, b]	73.4± 3.43 [a, b]	$F(3,29)=18.15$, $p<0.001$, $\eta^2=0.653$
Vmax ($km.h^{-1}$)	24.3± 1.9 *	29.2± 1.9	$F(1,31)=52.43$, $p<0.001$, $\eta^2=0.628$	23.2± 1.2	26.3± 1.3 [a]	28.1± 1.1 [a]	30.2± 1.8 [a, b, c]	$F(3,29)=45.27$, $p<0.001$, $\eta^2=0.824$
$S.min^{-1}$	0.02± 0.02*	0.11± 0.04	$F(1,31)=75.15$, $p<0.001$, $\eta^2=0.708$	0.01± 0.01	0.04± 0.02 [a]	0.08± 0.04 [a]	0.12± 0.02 [a, b]	$F(3,29)=51.77$, $p<0.001$, $\eta^2=0.843$
$A.min^{-1}$	0.6± 0.2*	0.8± 0.2	$F(1,31)=6.81$, $p=0.014$, $\eta^2=0.180$	0.5± 0.1	0.8± 0.2 [a]	0.8± 0.2 [a]	0.8± 0.1 [a]	$F(3,29)=57.56$, $p<0.001$, $\eta^2=0.439$
$HSR.min^{-1}$	0.8± 0.8*	3.5± 1.2	$F(1,31)=65.50$, $p<0.001$, $\eta^2=0.679$	0.3± 0.3	1.6± 0.8 [a]	2.7± 1.3 [a]	4.2± 0.6 [a, b]	$F(3,29)=48.73$, $p<0.001$, $\eta^2=0.834$
$Na.min^{-1}$	0.4± 0.1*	0.2± 0.1	$F(1,31)=67.46$, $p<0.001$, $\eta^2=0.685$	0.4± 0.1 [c, d]	0.4± 0.1 [c, d]	0.2± 0.1	0.2± 0.1	$F(3,29)=21.49$, $p<0.001$, $\eta^2=0.690$
DuelEFF (%)	88.7± 7.6	88.8± 6.5	$F(1,31)=0.001$, $p=0.973$, $\eta^2=0.000$	80.0± 8.3	89.9± 6.4	88.3± 7.5	89.2± 6.0	$F(3,29)=0.12$, $p=0.951$, $\eta^2=0.012$
TackleEFF (%)	69.6± 6.8*	51.9± 15.0	$F(1,31)=21.20$, $p<0.001$, $\eta^2=0.406$	67.8± 7.0 [d]	72.8± 5.7 [d]	59.8± 10.8	45.1± 15.0	$F(3,29)=11.36$, $p<0.001$, $\eta^2=0.540$
RuckEFF (%)	81.0± 6.5*	70.3± 10.3	$F(1,31)=13.44$, $p<0.001$, $\eta^2=0.302$	81.2± 5.5 [d]	80.5± 8.5	69.6± 12.2	79.9± 9.3	$F(3,29)=4.25$, $p=0.013$, $\eta^2=0.305$

* significantly different from backs ; *a* significantly different from front row and lock ; *b* significantly different from back row ; *c* significantly different from inside backs; *d* significantly different from outside backs, for p<0.05. $D.min^{-1}$, distance per minute, $S.min^{-1}$, number of sprints per minute, $HSR.min^{-1}$, high-speed running per minute, Vmax, maximum velocity, $A.min^{-1}$, number of accelerations per minute, $Na.min^{-1}$, number of combat tasks per minute.

Player activity data in match game according to position

In competition, distance per minute ($D.min^{-1}$) was higher in the backs than in the forwards ($p < 0.001$; d=2.40). They also performed more than four times as many sprints per minute ($p < 0.001$; d=3.09), 24.6% more accelerations per minute ($p = 0.014$; d=0.93), reached a higher maximum velocity (+4.9 $km.h^{-1}$) ($p < 0.001$; d=2.58) and covered more than three times as much high-speed running distance per minute as the forwards ($p < 0.001$; d= 2.88).

Specifically, inside backs and outside backs had a significantly higher $D.min^{-1}$ than front row and lock and back row (73.5 ± 5 $m.min^{-1}$ and 73.4 ± 3 $m.min^{-1}$ vs. 62.8 ± 4 $m.min^{-1}$ and 66.6 ± 3 $m.min^{-1}$ respectively) (all $p < 0.03$; all $d>1.77$). Maximum velocity reached in all groups was higher than in front row and lock. In addition, the outside backs had a higher velocity (V_{max}) than the back row and inside backs ($p < 0.001$; d= 2.47 and p = 0.04; d= 1.39, respectively). The number of sprints ($S.min^{-1}$) was lower in front row and locks compared to all other groups (-75%, p =0.002; d= 2.62 compared to back row, -87.5%, $p < 0.001$; d= 3.31 compared to inside backs and -91.7%, $p < 0.001$ compared to outside backs). Outside backs also performed more $S.min^{-1}$ than back row ($p < 0.002$; d= 4.51). The same differences between groups were observed for $HSR.min^{-1}$. Concerning the number of accelerations, back row (+45.3%; d= 2.00), inside backs (+45.3%; d= 1.76) and outside backs (+41.5%; d= 2.14) performed more accelerations than front row and locks (all $p < 0.04$).

In contrast, forwards performed a significantly higher number of fighting actions per minute (Table 2, +94.7%, $p < 0.001$; d= 2.93) and were more efficient in rucks (p= 0.002; d= 1.3) and tackles (p= 0.003; d= 1.64) than backs (Table 2). Specifically, more fighting actions per minute were achieved in front row and lock and back row (both 0.37± 0.06 $na.min^{-1}$) compared to inside backs (0.17± 0.05) and outside backs (0.20 ± 0.08) (all $p < 0.005$, all $d > 2.38$). Ruck efficiency was 10.3% higher in front row and locks compared to outside backs ($p < 0.02$; d= 1.47), and both groups of forwards had a higher tackle efficiency than outside backs (a difference of 22.7%, d= 2.15 for front row and locks, and 27.7%, d= 2.39 for back row, all $p < 0.001$).
